# Physical and occupational therapy service delivery models for populations identified as hard-to-reach: A scoping review

**DOI:** 10.1371/journal.pone.0310993

**Published:** 2024-11-13

**Authors:** Kenneth S. Noguchi, Muhib Masrur, Lori Letts, Susanne Sinclair, Sarah Wojkowski, Julie Richardson

**Affiliations:** 1 School of Rehabilitation Science, McMaster University, Hamilton, Canada; 2 Department of Health Research Methods, Evidence and Impact, McMaster University, Hamilton, Canada; PLOS: Public Library of Science, UNITED KINGDOM OF GREAT BRITAIN AND NORTHERN IRELAND

## Abstract

**Background:**

The delivery of rehabilitation services for hard-to-reach populations (e.g., refugees) is highly complex. There is a need for evidence-based approaches to deliver physiotherapy (PT) or occupational therapy (OT) services to this underserved group.

**Objectives:**

The purpose of this scoping review was to identify PT and OT service delivery models that have been implemented, for populations typically identified as hard-to-reach and their associated health outcomes.

**Eligibility criteria:**

Articles were eligible if they described PT and/or OT services for hard-to-reach populations. There were no restrictions on study design.

**Study selection:**

Six electronic databases (AMED, CINAHL, MEDLINE, EMBASE, Healthstar, and PsycINFO) were searched from January 2000 to June 2023. Articles were screened in duplicate by two independent reviewers, and conflicts were resolved by consensus.

**Results:**

Twenty-one articles with variable sample sizes (min, max n = 3 to 237) were included and detailed PT and/or OT services for immigrants/migrants, refugees, hard-to-reach veterans, people experiencing homelessness, lower incomes, trauma/torture, and those living in rehabilitation-deficient areas. Common rehabilitation needs (e.g., clinician to client connectivity), barriers (e.g., high transportation costs) and facilitators (e.g., encouragement) were identified among the various populations, mainly due to intersecting identities such as those who are both traumatized and refugees. Unique factors pertaining to the PT and OT services were also identified in some groups, including access to child and family services for people experiencing homelessness.

**Conclusions:**

Despite common and individual needs, barriers, and facilitators in hard-to-reach groups in the literature, there is a need for studies with larger sample sizes, rigorous methodology and a conscious effort to publish the results of interventions to generate stronger recommendations for practice.

## Introduction

Rehabilitation is defined as “a set of interventions designed to optimize functioning and reduce disability in individuals with health conditions in interaction with their environment” [1]. As the population ages, there is likely to be an increased demand for rehabilitation, due to a concomitant increase in the prevalence of chronic conditions and disability. Although rehabilitation is an important part of universal health coverage, the need for rehabilitation is largely unmet [2]. The need for rehabilitation increased by 63% between 1990 and 2019, which is mainly attributed to the prevalence of persons with chronic conditions [3]. This is especially true in the Canadian context, wherein 1 in 4 Canadians will be >65 years by 2023, and the population of seniors is projected to reach close to 25% of the overall population by 2040 [4]. Canadians with chronic conditions also account for over 70% of all nights spent in hospital and 50% report moderate to severe disability in daily living [5,6]. Thus, optimizing and preserving physical functioning is a key priority for Canadians living with chronic illness.

It is widely understood that people with chronic conditions have unmet rehabilitation needs. A recent scoping review found that fewer than 35% of people in North America who need rehabilitation access these services [7]. Moreover, ‘hard-to-reach’ populations, such as those experiencing homelessness, are disproportionately impacted by unmet healthcare needs [8]. Increasing access to rehabilitation services for hard-to-reach populations can be highly complex due to issues related to the social determinants of health such as low socioeconomic status, lack of housing, decreased social support, unhealthy behaviors, and inequitable access to health services [9]. In part due to its complexity, there has been a paucity of research to examine rehabilitation services available to hard-to-reach populations. A recent review examined organisational strategies to expand access of rehabilitation services to community dwelling persons, and found that only five studies examined interventions to improve equitable access for underserved populations [10].

Physiotherapists (PTs) and occupational therapists (OTs) can mitigate the resulting health care system burden by using their expertise in optimizing function. They can also employ innovative methods specifically developed to address the unmet need for rehabilitation services among individuals facing economic, social, and geopolitical barriers. An example of such initiatives includes the MAC H^2^OPE clinic (Helping Hamiltonians through Occupational Therapy and Physiotherapy Engagement) which was established in 2013 to provide rehabilitation supports to persons who otherwise had no access to PT and OT services in Hamilton, Canada. Initially services were provided, by leveraging clinical placements of students in the McMaster Master of Science (MSc) Physiotherapy and Occupational Therapy programs. However, prior to the start of the COVID-19 pandemic, two local primary care teams (known as Family Health Teams) facilitated outreach by staff occupational therapists and physiotherapists, which enabled the MAC H^2^OPE Clinic to provide OT and PT services continuously, with extra appointment times available when students were on placement. During the COVID-19 pandemic lockdown, services at the clinic were suspended, and were subsequently subsumed by local primary care teams. Regardless of the delivery location or source of service provision, it is important for rehabilitation services to be tailored to reach those in the community most in need. Although some evidence has been presented for specific population groups and among individual research studies, no review has synthesized the evidence for OT and PT services in a broader range of hard-to-reach populations.

This scoping review was undertaken to identify rehabilitation service delivery models that have been implemented, and their associated health outcomes for populations typically identified as hard-to-reach. Considering a goal of this research is to identify the current breadth and depth of the literature, and the emerging nature of this topic, a scoping review is an appropriate methodology for this investigation [11].

## Methods

### Protocol and registration

This review followed the methodological framework proposed by Arksey & O’Malley [11], the Joanna Briggs Institute [12], Levac, Colquhoun & O’Brien [13], as well as Peters and colleagues [14]. In general, the proposed stages for the review included: identifying the research question, publishing a protocol, identifying relevant studies, selecting studies for detailed analysis, extracting and charting data, and collating, summarizing, and reporting the results. The review protocol was published before data extraction [15], and this manuscript is reported in accordance with the PRISMA Extension for Scoping Reviews (PRISMA-ScR).

### Eligibility criteria

Articles were eligible for inclusion in this review if they discussed the delivery of PT and OT services to adults (≥18 years) considered to be members of hard-to-reach populations. In 2022, the Centers for Disease Control recommended replacing the term hard-to-reach with ‘people who are underserved by [a specific service]’, meaning that they would have limited access to services that are accessible, acceptable, and affordable, including healthcare. However, they also state that underserved should not be used when certain groups are disproportionately affected, which is the case with the hard-to-reach populations. For this reason and to be consistent with our search strategy we have kept the term hard-to-reach throughout. Hard-to-reach populations were defined *a priori* and included: People who are/were experiencing homelessness, incarceration, alcohol or drug use disorders, immigrants or migrants, refugees, medically uninsured individuals, people with lower incomes, sex workers, sex and gender minorities (e.g., transgender and two-spirit individuals), people who have experienced trauma or torture, and veterans. There were no restrictions on study design, which included experimental and quasi-experimental study designs (e.g., randomized, and non-randomized controlled trials), observational studies (e.g., retrospective cohort), systematic and scoping reviews, descriptive observational studies (e.g., case report), and qualitative studies. There were no restrictions on geographical context.

### Information sources and search strategy

Six electronic databases (AMED, CINAHL, MEDLINE, EMBASE, Healthstar, and PsycINFO) were initially searched from January 2000 to April 2018 for articles using PT and/or OT services for people underserved by rehabilitation. The search was subsequently updated in June 2021 and June 2023. Reference lists from relevant articles were also hand-searched. The full search strategy for MEDLINE is detailed in the study protocol [15]. In brief, the search included keywords and subject headings for hard-to-reach populations, as well as PT and OT. We did not search for specific population groups without search terms related to hard-to-reach or social disadvantage, since it is possible that some eligible populations may not be considered hard-to-reach. The search was limited to articles published in English since this is the only language spoken by all members of the research team, and therefore was applied to prevent inaccurate translation of research due to not having social or cultural context if automated translation supports were used [16].

### Selection of sources

All potentially eligible articles were uploaded to two systematic review management programs (Initial search, April 2018: Rayyan, Qatar Computing Research Institute, BKU, Doha, Qatar; Updated searches, June 2021 and 2023: Covidence, Veritas Health Innovation Ltd, Melbourne, Victoria, Australia). Five titles and abstracts, and full-text articles were randomly selected to pilot the screening process with all members of the research team. There were no conflicts during the pilot process with all members of the research team in agreement about inclusion and exclusion decisions. After duplicates were removed, two independent pairs of reviewers (KSN, MM, LL, LS, SS, JR, SW, HK) screened titles and abstracts, and full-text articles. Disagreements between pairs of reviewers were addressed via consensus discussion or consultation of a third reviewer who was not involved in the disagreement.

### Data extraction and synthesis

Two independent pairs of reviewers piloted the data extraction process using the published extraction form to ensure consistency [15]. Briefly, the form included an eligibility checklist, data on study design, participant demographic information, theoretical frameworks used in interventions, delivery format (e.g., face-to-face vs online, group vs individual), the role of the PT or OT, and whether the article describes the PT or OT needs of people underserved by rehabilitation. The only changes to the published form were the addition of race/cultural identity and geographical location. Data was then extracted by single reviewers (KSN, MM) for feasibility purposes. Any conflicts or concerns were resolved by consensus or by a third reviewer (JR, LL).

Included studies were categorized based on population: people who are/were incarcerated; immigrants or migrants; refugees; experiencing homelessness; with lower incomes; alcohol or drug use disorders; have experienced trauma or torture; and veterans. Extracted data were presented in tabular format to include study descriptions, theoretical framework utilized (if any), intervention description, specialty delivering intervention (PT, OT), service delivery model, PT/OT roles, barriers and facilitators of service delivery, and health outcomes. A narrative summary of the charted data was also provided to describe how study results related to the review’s objectives.

## Results

**Fig 1:**
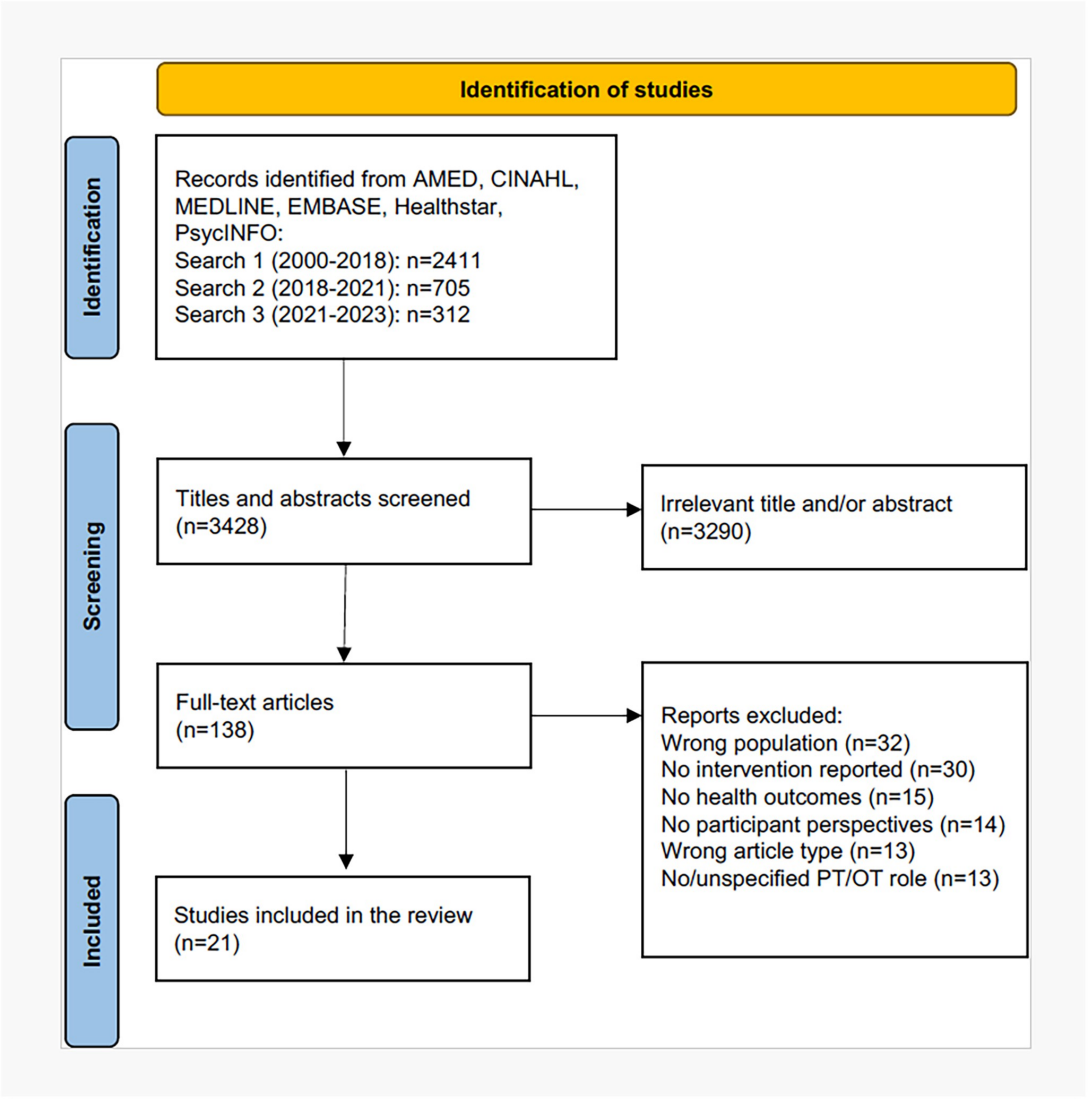
PRISMA-ScR flow diagram.

The search yielded 3,498 records after deduplication, from which 21 met the inclusion criteria based on full text review (Fig 1).

Study descriptions, including their samples, study designs, intervention summaries, and roles of the PT or OT have been summarised in Table 1. Sample sizes from individual studies ranged from 3 [17] to 237 participants [18], and included individuals who are/were incarcerated, immigrants or migrants, refugees, experiencing homelessness, people with lower incomes, disabilities in rehabilitation-deficient areas, alcohol or drug use disorders, people who have experienced trauma or torture, and veterans in rehabilitation deficient (rural) areas. There were three systematic or scoping reviews, which included 7 to 178 studies [19–21].

**Table 1:** Included study information (N = 21 studies).

Study	Country	N	Age	Sex (n, %F)	Delivery		Study design
					PT	OT	
***Immigrants or migrants (N = 2 studies)***							
Gupta 2012* [22]	US	NR	NR	NR		X	Mixed-methods program evaluation
Thulstrup 2021† [23]	DE	27	51.1 (46.6 to 55.7)	n = 26 (100%)	X		Pre-post mixed methods
***Refugees (N = 1 study)***							
Madsen 2015* [17]	DE	3	26 to 50	n = 1 (33%)	X		Case series
***Experiencing homelessness (N = 5 studies)***							
Boisvert 2008* [24]	US	18	19 to 62	NR		X	Pre-post mixed methods
Gutman 2004* [25]	US	26	23 to 58	n = 26 (100%)		X	Quasi-experimental
Thomas 2011* [21]	N/A	7 studies	NR	NR		X	Systematic review
Chapleau 2012* [26]	US	57	I: 47.1 ± 12.3 C: 45.5 ± 9.2	n = 25 (44%)		X	Non-randomized controlled trial
Roy 2017* [20]	N/A	178 studies	NR	NR		X	Scoping review
***Lower incomes (N = 5 studies)***							
Barnes 2008* [27]	US	19	25 to 59	n = 12 (63%)		X	Single group pre-post
Gilstrap 2013* [28]	US	64	51.3	n = 64 (100%)	X		Prospective cohort
Ciro 2015* [29]	US	11	65.3 ± 8.9	n = 7 (64%)		X	Quasi-experimental
Towfighi 2020† [30]	US	100	58 ± 9	n = 71 (71%)		X	Pilot RCT
Rahmati 2023‡ [19]	N/A	7 studies	65 to 79	NR		X	Systematic review
***Experienced trauma or torture (N = 3 studies)***							
Palic 2009* [31]	DE	26	39.0 ± 9.9	n = 12 (46%)	X		Single group pre-post
Stade 2015* [32]	DE	9	47.3 ± 5.8	n = 5 (56%)	X		Single group pre-post
Dibaj 2017* [33]	NO	6	30 to 60	n = 1 (17%)	X		Case series
***Veterans described as hard-to-reach (N = 1 study)***							
Levy 2015* [34]	US	26	69% ≤64 yrs	n = 2 (8%)	X		Retrospective pre-post
***Clients with disabilities in rehabilitation-deficient areas (N = 3 studies)***							
Carr 2005* [18]	UK	237	I_1_: 42.5 ± 11.2 I_2_: 42.0 ± 10.6	n = 143 (60%)	X		RCT
Morita 2009* [35]	JP	69	I: 69.3 ± 10.9 C: 69.6 ± 9.8	n = 29 (42%)	X		Prospective survey
Cook 2003* [36]	UK	37	53.0 ± 11.6	n = 7 (19%)		X	Quasi-experimental
***Drug or alcohol use disorders (N = 1 study)***							
Ussher 2000† [37]	UK	10	37.4	n = 3 (30%)		X	Case series

US = United States, DE = Denmark, NO = Norway, UK = United Kingdom, JP = Japan, N/A = Not applicable, NR = Not reported, RCT = Randomized controlled trial.

All data extraction completed by KSN and MM.

Date data extracted:

* October 2018;

^†^ June 2021;

^‡^ June 2023.

### Immigrants or migrants

#### Intervention characteristics

Two studies [22,23] included immigrants or migrants and all interventions were delivered face-to-face by OTs or PTs. Gupta (2012) [22] described an OT service delivery model and used a mixed-methods program evaluation. The OT-based program was delivered to new immigrants alongside their English Language Learner classes. Program modules addressed cultural transition, self-efficacy and wellbeing, cultural beliefs about time and time use; stress and impact in health; and workplace norms, stress, and injury. They instructed participants on new ways of performing daily activities, initiating community integration, and navigating the complexities of their new society. Using a combination of qualitative and quantitative analyses, they assessed the program’s effectiveness on self-efficacy, routines and schedules, time management, stress, sleep, injury, and overall health. Thulstrup and colleagues (2021) [23] described the feasibility and effects of a twelve-week, twice weekly, PT intervention for immigrant women in Denmark using a pre-post study design. The exercises were individualized to preferences of the women, and consisted of endurance, strength, coordination, balance, and resistance exercises. There were two program locations, each offering privacy through closed curtains, locked doors, and no video surveillance, which allowed the women to remove clothing and/or headscarves as needed.

#### Needs of the population

The women in the study by Thulstrup and colleagues (2021) reported needing assistance with planning exercises, exercise variability, a safe space for women to remove head coverings, and quiet music to hear instructions from the instructor. Their expectations specifically for the program included pain reduction, weight loss, and general wellbeing [23].

#### Effects on health outcomes

Gupta (2012) identified that motivation for self-improvement, group discussions and educational materials were facilitators to delivery [22]. Conversely, Thulstrup et al (2021) found that the PTs’ explanation of theoretical underpinnings behind the exercises was a barrier to engagement [23]. Collectively, there were no significant changes in body mass index, fat percentage, walking performance, lower-body strength, or quality of life as a result of the PT intervention [23]. However, they also noted 60% of women had improved walking performance and BMI, and 50% of women had improved lower-body strength [23]. Qualitatively, the participants in the OT intervention reported improvements in stress, sleep, injury, overall health, healthy food choices, physical activity participation and awareness of work-related injuries [22].

### Refugees

#### Intervention characteristics

Only one study [17] reported on a refugee population in Denmark. In this study, PTs delivered basic body awareness training (BBAT) in a hospital once weekly for one hour, over thirteen weeks. Participants also received usual care involving pharmacological treatment, psychoeducation, and cognitive behavioral therapy, which were delivered by other healthcare professionals.

#### Needs of the population

The qualitative interviews identified several needs for service delivery. Participants placed importance on the PTs being gentle, calm, empathetic, and catering to individual needs. Moreover, the PTs’ ongoing involvement with demonstrations helped participants learn about the movements. Finally, participants emphasized that contact with another person was essential for treatment [17].

#### Barriers and facilitators to implementation

In some cases, there were language barriers or cultural differences, which presented as a barrier to delivery, while regularity with the program was a facilitator [17].

#### Effects on health outcomes

Participants expressed that BBAT relieved pain and muscular tension, positively affected emotional state, and made it easier to sleep. Participants reported BBAT as being helpful when coping with stress and developing positive relationship with themselves and others [17].

### Individuals experiencing homelessness

Five studies [20,21,24–26] with various study designs (pre-post mixed methods [24], quasi-experimental [25], systematic review [21], scoping review [20], non-randomized controlled trial [26]) were conducted with people experiencing homelessness.

#### Intervention characteristics

Three primary studies [24–26] examined the impacts of OT interventions twenty-four weeks to twelve months in duration. All of the interventions were provided face-to-face and in group settings, with two of the studies [25,26] also having an individualized component. Across the interventions [24–26], the OTs mainly facilitated education sessions, which included various topics such as safety planning; drug and alcohol awareness; safe sex practices; assertiveness and advocacy skill training; anger management; stress management; boundary establishment and limit setting; vocational and educational skill training; money management; housing application; leisure exploration (e.g., relaxation, crafts, gardening); hygiene; medication routine; and nutrition and exercise. The OTs also provided handouts and led group discussions to educate the community on peer-recovery communities, and visited group meetings to observe patterns of interaction, and to provide ongoing support, guidance, and encouragement to participants [24]. In the systematic and scoping reviews [20,21], there were a broad range of interventions. The systematic review [21] included seven studies which examined the effectiveness of OT programs or identified the occupational needs of people experiencing homelessness, while the scoping review included 178 studies that described current and potential occupation-based practices. For details regarding the specifics of those interventions, we refer readers to the respective reviews [20,21].

#### Needs of the population

The systematic review by Thomas et al (2011) [21] identified money management, coping skills, employment and education, and leisure activities were important and common needs of people experiencing homelessness in the included studies, which align with the findings by Gutman et al (2004). Similarly, the scoping review found employment and education interventions, physical rehabilitation services, and child and family services were critical needs for people experiencing homelessness. Gutman et al (2004) also found interventions should be client-centred, and that leisure exploration exercises, knowledge of available resources, and developing assertiveness and advocacy skills were valuable. Participants in the intervention by Boisvert et al (2008) reported needing trust, respect, honesty, openness, helpfulness, leadership, integrity, willingness, and sobriety in their program.

#### Barriers and facilitators to implementation

In the scoping review by Roy et al (2017) [20], past negative experiences with service providers were identified as barriers to delivery, but that building rapport and sensitivity to traumatic experiences were facilitators. There were two main barriers to service delivery among the individual studies. Chapleau et al (2012) [26] found case managers felt OT referrals were not necessary once they were satisfied with their clients’ improvement. Gutman et al (2007) [25] identified that vulnerable populations are often not allocated the most appropriate services due to non-disclosure by the clients, which may present as a barrier to delivery. Gutman et al (2007) [25] also noted therapist support was helpful for safety planning. Similarly, Boisvert et al (2008) [24] also found that encouragement and a sense of support or community was a facilitator for client retention.

#### Effects on health outcomes

Roy et al (2017) also found reductions in trauma symptoms and increases in physical health among sports-based community building initiatives [20]. Among individual studies, there were no long-term differences in goal attainment [26], while shorter-term improvements in goal attainment were observed [24–26]. There was also improvements in social support and quality of life as a result of interventions [24]. Chapleau et al (2015) reported lower variance from ideal housing status over the twelve-month period compared to the control group. Boisvert et al (2008) [24] reported varying effects on health outcomes, and reduced risk of drug or alcohol relapse.

### Individuals with lower income

Five studies [19,27–30] included individuals with lower income. Three studies used an interventional design (e.g., single group pre-post [27], quasi-experimental [29], or pilot randomized controlled trial [30]), while two studies [19,28] reported service delivery models.

#### Intervention characteristics

The three interventions [27,29,30] occurred once or twice per week for one to two hours, over six to twelve weeks, delivered face to face by OTs in group settings, and mainly involved education sessions. Barnes et al (2008) implemented a lifestyle redesign program consisting of twelve educational modules involving: occupations, stress management, relationship, energy conservation and time management, exercise, joint protection, transportation/low-cost activities, nutrition, and spirituality/aging [27]. Ciro and Smith (2015) engaged in group education sessions that included: (1) didactic lessons on the benefits of meaningful activities; (2) exploring individually meaningful activities targeting physical, cognitive, and mental health; and (3) problem-solving environmental barriers that hinder participation in meaningful activities [29]. Towfighi et al (2020) reported on OTs implementing individualized nutrition, physical activity, and self-management action plans with participants after creating short term goals. Participants engaged in OT-led group sessions including didactic presentations of lifestyle practice, problem-solving strategies targeting barriers to identified goals, and engaged in group exercise and grocery purchasing activities [30].

The two studies about service delivery models were a prospective cohort study [28] and a systematic review [19]. The prospective cohort study followed individuals involved in a group and individual lifestyle modification program in a community health centre and over the phone for two years, led by a multidisciplinary team of PTs, physicians, nutritionists and health coaches in the United States. The systematic review by Rahmati and colleagues (2023) examined the effectiveness of a Community Aging in Place-Advancing Better Living for Elders (CAPABLE) program in seven studies. The CAPABLE program involved ten in-home sessions over six months and used psychosocial, behavioural, and biological factors that can impact overall health. Specifically, referenced program included an assessment, education, and a barrier to function identification process. OTs were involved in six sessions, while nurses were involved in four sessions. A program ‘handyman’ helped make home modifications based on the assessment findings and OTs recommendations [38]. In the systematic review, the included studies using the CAPABLE programs highlighted several hazard complaints of lower income individuals such as a lack of adaptive equipment, tripping hazards, clutter, and lighting [19].

#### Needs of the population

Participants in the study by Ciro and Smith (2015) [29] expressed a desire to participate in meaningful activities such as socialization, and preferred information on chronic disease management.

#### Barriers and facilitators to implementation

Across the studies, participants identified the following as environmental barriers to engaging in meaningful activities: intervention components not aligning with personal interests, low income, lack of awareness of available opportunities, lack of accessible transportation, functional limitations [29]. The studies also mentioned time-constraints and competing priorities as main reasons for attrition from interventions [28,30], and those with limited education and/or were immigrants cited cultural, financial, and education as barriers [28]. The studies also identified that availability of desired activities [29], group settings [30], and use of incentives [30] were facilitators to ongoing participation.

#### Effects on health outcomes

There were no quantitative changes in any health outcomes for the intervention studies, but one study reported increased life satisfaction through developing better social skills, increased knowledge of nutrition, and improved interpersonal skills [27]. Another study reported increases in meaningful participation in activities [29]. For the service delivery models, the program by Gilstrap et al (2013) yielded several health benefits and behaviours such as reduction of metabolic syndrome prevalence, lower rates of hypertension, increases in HDL cholesterol and lower glycated hemoglobin, anxiety and depression levels [28]. The review by Rahmati et al (2023) [19] also found the CAPABLE programs led to reductions in home safety hazards because of the service, as well as improvement in ADLs, IADLs, depression, falls efficacy, pain, and quality of life.

### Individuals who have experienced trauma or torture

#### Intervention characteristics

Three studies [31–33], two single group pre-post studies [31,32] and one case series [33], used PT interventions to improve health outcomes for people who have experienced trauma or torture. Two studies [31,32] used body awareness therapy administered face-to-face by PTs once per week for fourteen to eighteen weeks. The intervention by Stade and colleagues (2015) focused on relaxation and free movement [32], while the body awareness therapy provided by Palic et al (2009) was in conjunction with coping education and psychological therapy using cognitive behavioural theory [31]. The psychological therapy specifically involved methods to treat panic attacks, trauma hierarchies, in vitro exposure (through memory), breathing exercises, and anxiety/fear coping strategies. Trauma therapy was also employed in this study using principles of stabilization, coming to terms with trauma, and integration of traumatic memories and grieving using Herman’s model. In the case study by Dibaj et al (2017) [33], participants received 20 sessions of Narrative Exposure Therapy (NET) which consisted of the participants identifying and labelling traumatic experiences in their lives during the first session. In subsequent sessions, participants and therapists read the life narratives and applied prolonged exposure therapy applied for traumatic memories. Participants also received ten individualized PT sessions lasting one hour each.

#### Needs of the population

Stade et al (2015) [32] identified rehabilitation needs that may be culturally specific. They found that Arabic women in their study would feel uncomfortable in mixed-gender sessions and a need for an interpreter during the intervention period. Dibaj et al (2017) [33] found a need for family support during the program and assistance with current stressors.

#### Barriers and facilitators to implementation

Participants and therapists in the case series identified lack of motivation, low physical functioning, and concomitant conditions (e.g., pain) that lead to social isolation as barriers to benefitting from the intervention [33]. Participants in the study by Stade and colleagues (2015) identified physical disability, social anxiety with group therapy, lack of accessible transportation, and memory impairment leading to transport difficulties as being barriers to program participation [32].

#### Effects on health outcomes

Stade et al (2015) [32] identified statistically significant improvements in several health outcomes such as body awareness, somatization symptoms, self-reported disability post intervention. The interventions by Palic et al (2009) [31] resulted in reductions in post-traumatic stress disorder (PTSD) symptoms and other psychiatric symptoms, but no changes in cognitive function. In the case series by Dibaj et al (2017) [33], 50% of participants did not meet the clinical criteria for PTSD diagnosis by three months, and two participants achieved a clinically significant decrease in PTSD symptoms. Moreover, some participants were no longer diagnosed with depression at follow-up, or achieved clinically significant reductions in depressive symptoms or pain intensity.

### Veterans described as hard-to-reach

#### Intervention characteristics

Only one study [34] involved veterans who were hard-to-reach. Using a retrospective cohort design, PTs in the study delivered approximately fifteen virtual (74%) and face-to-face (26%) rehabilitation sessions over 100 days (approximately once per week) to veterans living in rural areas of the United States. The combination of in-person rehabilitation and telerehabilitation services included occupational, physical, and recreational therapies, psychological and nursing care from the patient’s home. Types of physical therapy provided included a general strengthening program, a lumbar and scapular stabilization program.

#### Effects on health outcomes

While no specific needs, barriers or facilitators were highlighted, the program resulted in improved physical and cognitive function, health-related quality of life, functional independence, and participants were satisfied with the program.

### Clients with disabilities in rehabilitation-deficient areas

Three studies [18,35,36] examined rehabilitation programs in clients with disabilities living in rehabilitation-deficient areas. Of these, two studies [18,36] used experimental designs (randomized controlled trial [18] and quasi-experimental [36]) and one was a prospective survey of a service delivery model [35].

#### Intervention characteristics

The PTs in the RCT by Carr et al (2005) [18] delivered a one-hour physical fitness class (termed ‘Back to Fitness’) twice weekly for four weeks involving aerobic, resistance and flexibility exercises, in addition to relaxation techniques using a cognitive-behavioural approach. The control group received individualized therapy of variable frequency and duration according to the patients’ needs, at the discretion of the PT. In contrast, the intervention reported by Cook et al (2003) [36] provided individualized OT, expanded general practitioner services, and care management for up to twelve months in a primary care setting. The OT had many roles including assessments, treatment planning, activity selection (e.g., personal hygiene, budgeting, adult literacy), and skill development. Participants also received a psychological intervention which included supportive counselling and anxiety management. Finally, Morita and colleagues (2009) conducted a prospective survey of a rehabilitation service over nearly seven years, implemented in a rehabilitation-deficient area in Japan [35]. A multidisciplinary team of PTs, nurses, care managers and social workers provided home-visit rehabilitation to improve physical function, mobility, and housing environmental modifications.

#### Barriers and facilitators to implementation

Cook et al (2003) [36] identified there was a high annual cost for the program due to continuation of referral and therapy, representing a potential barrier for individuals. There were no other needs, barriers and facilitators highlighted for clients in rehabilitation deficient areas.

#### Effects on health outcomes

The RCT by Carr et al (2005) [18] found no significant differences in change scores between groups for quality of life, disability, and pain self-efficacy. However, the OT intervention by Cook et al (2003) resulted in greater social functioning and improvements in psychiatric symptoms including anxiety, depressed mood, incongruity, overactivity, and pressure of speech (i.e., symptoms of affect); incoherence or irrelevance (i.e., symptoms of schizophrenia). Moreover, there were significant improvements in most domains of the Health of the Nation Outcome Scale, except for the physical illness and disability, and non-accidental self-injury domains [36]. Finally, over a mean intervention period of approximately twelve months, Morita et al (2009) observed significant improvements in ADL disability compared to a reference group of people who did not receive rehabilitation [35].

### Individuals with drug or alcohol use disorders

#### Intervention characteristics

Only one study [37] focused on individuals with drug or alcohol use disorders using a qualitative case series design. Ussher et al (2000) assessed the effectiveness of an OT-delivered physical activity intervention in a community-based alcohol service, with support from an exercise professional. The 6-week, face-to-face, group-based program aimed to promote independence, integration, education, and health among clients. Specifically, it involved twenty-five minutes exercise counselling, fifty minutes supervised exercise, fifteen minutes client feedback. Exercise counselling involved cognitive-behavioural techniques to promote exercise adherence. The intervention used goal setting, self-monitoring, decision-making, and relapse prevention techniques. Discussion topics included the role of exercise and lifestyle PA, importance of fitness for ADLs, associations between social PA and heavy social drinking, and motivation to a PA program.

#### Effects on health outcomes

Ussher et al (2000) [37] found attending exercise classes resulted in reduced attendance due to problems at home or poor health. However, they also found a tailored home exercise program and support early in the program provided motivation to exercise. Increased confidence, positive conversations around exercise instead of drinking, and group discussions were also recognized facilitators. Although there were mixed attendance rates for the program, more than 50% of goal attainment was reported in 9 of 15 participant goals. There were general improvements in health and motivation, and positive attitudes towards the program.

## Discussion

People underserved by rehabilitation are also underrepresented in research. This scoping review aimed to identify rehabilitation service delivery models that have been implemented, and their associated health outcomes for populations typically identified as hard-to-reach, across a broad range of studies. We have identified several needs for the programs and groups of people, barriers, and facilitators to implementation, and highlighted potential health benefits of these models for several populations with respect to rehabilitation services. Although each population has their own unique needs, barriers, facilitators, and results on health outcomes, several commonalities were present across the different groups.

There were common service delivery needs for hard-to-reach populations related to rehabilitation. First, there was a clear need for client education, assistance and support to complete rehabilitation programs among immigrants and migrants [23], people experiencing homelessness [24,25], lower-income individuals [29], and individuals who have experienced trauma or torture [33]. Moreover, people underserved by rehabilitation identified a need for clinicians to be connected to their clients. For example, clinicians delivering rehabilitation services should be empathetic and gentle [17], build rapport and social sensitivity toward their clients [20], and continue to be culturally aware in sessions [17,32].

Populations also experienced common barriers and facilitators to participation in rehabilitation. For instance, a sense of community or group programming were facilitators for ongoing participation in lower income individuals [30] and people experiencing homelessness [24], and support from clinicians, such as assistance with movement patterns or encouragement, was helpful for refugees [17] and for people experiencing homelessness [24]. There were also shared barriers among groups, including cultural differences in refugees [17] and lower income individuals [28], as well as barriers to access (e.g., transportation, cost) and physical disabilities in traumatized refugees [32,33] and lower income individuals [29].

The overlap in these populations’ needs, barriers and facilitators could be due to intersections in identity. For example, two studies [31,32] included traumatized refugees, who experience needs that are aligned with both refugees and individuals who have experienced trauma. Similarly, people experiencing homelessness may also have low incomes due to low employment rates [39], and therefore expressed similar barriers to engagement such as transportation access issues [29]. Despite some common needs, barriers, and facilitators among various people underserved by rehabilitation, it is important to recognize that each population group has their own unique experiences. For instance, people experiencing homelessness stated that money management [21] and access to child and family services [20] were unique needs for this group. Moreover, some aspects may be considered facilitators for some, but barriers for others. For example, while a sense of community was important for low-income individuals or those experiencing homelessness, group settings were barriers for refugees who had experienced trauma. Therefore, while intersecting identities may exist, researchers and clinicians should interpret the common observed themes across populations with caution and be mindful of the potential differences between groups of hard-to-reach populations related to needs for rehabilitation services.

The included studies collectively identified potential for improvement in a range of health outcomes, such as benefits in physical and psychological wellbeing. However, there is a need for rehabilitation professionals to publish the results of interventions with hard-to-reach groups. Many of these studies did not apply rigorous research designs to test the effectiveness of the implemented rehabilitation services. In fact, there were only two randomized controlled trials [18,30], and a range of other designs that quantitatively assessed the effects of the rehabilitation programs, which limits the strength of our conclusions. Arguably, rigorous research designs and approaches, such as RCTs, can be difficult to implement with these populations. Studies have found underrepresented groups are willing to engage in research, but experience barriers to full engagement such as mistrust or distrust in the healthcare system, or competing priorities (e.g., financial stability, family roles) [40], resulting in smaller samples and the use of more pragmatic designs, as shown in this review. Therefore, there is a critical need for rigorous controlled studies that promote inclusive clinical trial practices such as those recommended by the US Food and Drug Administration (FDA) [41] to increase the number of high quality research designs and representation of those for whom interventions are designed, and to better understand the effects of rehabilitation on health outcomes. For instance, the FDA report suggests broadening trial eligibility criteria and simplifying trial procedures, such as reducing the number of study visits or streamlining participant renumeration, to help studies achieve larger sample sizes, retain participants, and increase the generalizability of findings [41].

## Conclusion

PT and OT interventions and service delivery models have been successfully implemented in various populations identified as being hard-to-reach. This review identified several common needs, barriers, and facilitators experienced by these groups, but also unique experiences for each population. Despite the breadth of research covered in this scoping review, there is a need for rigorous studies with larger samples to better understand the effects of interventions or delivery models on health outcomes.

## Supporting information

S1 File Studies identified. (XLSX)

S2 File PRISMA-ScR checklist. (DOCX)

S3 File Medline search strategy. (DOCX)

S4 File Data extraction form. (DOCX)
