# Supplementary material for: Physical and occupational therapy service delivery models for populations identified as hard-to-reach: A scoping review
Source: PLoS One. 2024 Nov 13;19(11):e0310993. doi: 10.1371/journal.pone.0310993 (PMC11559991; doi:10.1371/journal.pone.0310993)
Supplement: S3 File — (DOCX) [file pone.0310993.s003.docx]

Search Set Ovid MEDLINE

1 physiotherap*.mp.

2 Physical Therapy Specialty/

3 physical therap*.mp.

4 Occupational Therapy/

5 occupational therap*.mp.

6 Vulnerable Populations/

7 vulnerable population*.mp.

8 HOMELESS PERSONS/

9 homeless*.mp.

10 Urban Population/

11 urban population*.mp.

12 inner city*.mp.

13 POVERTY/ or poverty.mp.

14 disenfranchise*.mp.

15 hard to reach population*.mp.

16 socially disadvantage*.mp.

17 alcoholics/ or criminals/ or disabled persons/emigrants and immigrants/ or

homeless persons/ or medically uninsured/ or refugees/ or sex workers/

sexual/gender minorities/transients and migrants/or vulnerable populations/

18 alcoholic*.mp.

19 criminal*.mp.

20 disabled person*.mp.

21 emigrant*.mp.

22 immigrant*.mp.

23 medically uninsured.mp.

24 refugee*.mp.

25 sex worker*.mp.

26 sexual minorities.mp.

27 gender minorities.mp.

28 transient*.mp.

29 migrant*.mp.

30 drug user*.mp. or Drug Users/

31 prisoner*.mp. or Prisoners/

32 1 or 2 or 3 or 4 or 5

33 6 or 7 or 8 or 9 or 10 or 11 or 12 or 13 or 14 or 15 or 16 or 17 or 18 or 19 or

20 or 21 or 22 or 23 or 24 or 25 or 26 or 27 or 28 or 29 or 30 or 31

34 32 and 33

35 limit 34 to (English and journal article)
