# Supplementary material for: Physical and occupational therapy service delivery models for populations identified as hard-to-reach: A scoping review
Source: PLoS One. 2024 Nov 13;19(11):e0310993. doi: 10.1371/journal.pone.0310993 (PMC11559991; doi:10.1371/journal.pone.0310993)
Supplement: S4 File — (DOCX) [file pone.0310993.s004.docx]

**Scoping Review – Rehabilitation for Hard to Reach Populations**

| Reviewer’s Initials: | | | | | | | | | Date: |
| --- | --- | --- | --- | --- | --- | --- | --- | --- | --- |
| First author’s last name: | | | | | | | | | Year: |
| **Eligibility:** | | | | | | | | | |
| PT or  OT or  Both | | |  | | | | English language | | |
| Age ≥18 yrs. | | |  | | | | Vulnerable population | | |
| High income country (Specify): | |  | | | | | Model of service provision or intervention | | |
| **Article type:** | | | | | | | | | |
| Non-experimental design | | | Experimental design | | | | | | |
| Qualitative study  Editorial  Practice guideline  Review  Other: | | | Interventional study  Pre-post  Non-randomized trial  RCT  Other: | | | | Observational study  Cross sectional  Cohort  Other: | | |
| **Article details:** | | | | | | | | | |
| Population(s) | | | | | | | | | |
| Homeless | | | Offenders | | | | Economically disadvantaged | | |
| Immigrants | | | Refugees | | | | Medically uninsured | | |
| Migrants | | | Alcoholics | | | | Sex workers | | |
| Veterans | | | Drug users | | | | Sex and gender minorities | | |
| Other (Specify): | | | | | | | | | |
| Setting | Community (Specify): | | | | | | | | |
|  | Transitional care | | | | | Other (Specify): | | | |
| **Research Question/Purpose:** | | | | | | | | | |
|  | | | | | | | | | |
| **The paper describes an  Intervention or  Model of service delivery** | | | | | | | | | |
| Theoretical Framework or Model Yes  No  Details: | | | | | | | | | |
| **Intervention details:** | | | | | | | | | |
| Chronic disease management/self-management | | | | | Fall prevention | | | | |
| Pain management/self-management | | | | | Exercise program/prescription | | | | |
| Return to work | | | | | Mobility aid/equipment prescription | | | | |
| ADL training | | | | | Other: | | | | |
| Format/Delivery | | | | Delivered by | | | | Intensity, frequency, duration | |
| Face to face, individual  Face to face, group  Telephone  Online  Other: | | | |  | | | |  | |

| Additional details about the intervention | | | | |
| --- | --- | --- | --- | --- |
|  | | | | |
| **Model of Service Delivery details:** | | | | |
|  | | | | |
| **Describes the role of the OT/PT  Yes  No Please provide details.** | | | | |
|  | | | | |
| **Describes OT/PT needs of hard to reach populations?  Yes  No** | | | | |
| Physical health problem  Mental health problem  Navigating community resources/programs  General health/lifestyle issue | | | Referral to other health care providers  Referral to other sectors (Social services)  Other: | |
| **Describes facilitators and barriers to service delivery in hard to reach populations?  Yes  No** | | | | |
| Barriers: | | | Facilitators: | |
|  | | |  | |
| **Describes health outcomes used in hard to reach populations?  Yes  No Please list.** | | | | |
|  | | | | |
| **For experimental studies only** | | | | |
| Sample Size | Total | Int: | | Control: |
| Groups’ mean age | | Int: | | Control: |
| Sex | | Int: %F %M %Other | | Control: %F %M %Other |
| Results: | | | | |
|  | | | | |
